# Differences between motor execution and motor imagery of grasping movements in the motor cortical excitatory circuit

**DOI:** 10.7717/peerj.5588

**Published:** 2018-08-28

**Authors:** Hai-Jiang Meng, Yan-Ling Pi, Ke Liu, Na Cao, Yan-Qiu Wang, Yin Wu, Jian Zhang

**Affiliations:** 1 School of Kinesiology, Shanghai University of Sport, Shanghai, China; 2 School of Sports, Anqing Normal University, Anqing, China; 3 Shanghai Punan Hosptial of Pudong New District, Shanghai, China; 4 School of Economics and Management, Shanghai University of Sport, Shanghai, China

**Keywords:** Motor imagery, Motor-evoked potential, Motor execution, Transcranial magnetic stimulation, Motor cortex, Intracortical inhibitory and excitatory circuits

## Abstract

**Background:**

Both motor imagery (MI) and motor execution (ME) can facilitate motor cortical excitability. Although cortical excitability is modulated by intracortical inhibitory and excitatory circuits in the human primary motor cortex, it is not clear which intracortical circuits determine the differences in corticospinal excitability between ME and MI.

**Methods:**

We recruited 10 young healthy subjects aged 18−28 years (mean age: 22.1 ± 3.14 years; five women and five men) for this study. The experiment consisted of two sets of tasks involving grasp actions of the right hand: imagining and executing them. Corticospinal excitability and short-interval intracortical inhibition (SICI) were measured before the interventional protocol using transcranial magnetic stimulation (baseline), as well as at 0, 20, and 40 min (T0, T20, and T40) thereafter.

**Results:**

Facilitation of corticospinal excitability was significantly greater after ME than after MI in the right abductor pollicis brevis (APB) at T0 and T20 (*p* < 0.01 for T0, and *p* < 0.05 for T20), but not in the first dorsal interosseous (FDI) muscle. On the other hand, no significant differences in SICI between ME and MI were found in the APB and FDI muscles. The facilitation of corticospinal excitability at T20 after MI correlated with the Movement Imagery Questionnaire (MIQ) scores for kinesthetic items (*Rho* = −0.646, *p* = 0.044) but did not correlate with the MIQ scores for visual items (*Rho* = −0.265, *p* = 0.458).

**Discussion:**

The present results revealed significant differences between ME and MI on intracortical excitatory circuits of the human motor cortex, suggesting that cortical excitability differences between ME and MI may be attributed to the activation differences of the excitatory circuits in the primary motor cortex.

## Introduction

Functional activation of the human motor cortex is associated with not only motor execution (ME) but also motor imagery (MI). Previous studies using transcranial magnetic stimulation (TMS) have reported that both activities enhance the excitation of the human primary motor cortex (Wang et al., 2014; Bisio et al., 2017). Furthermore, many studies have consistently shown that motor cortical excitability is significantly smaller during MI than during ME (Avanzino et al., 2015; Bonassi et al., 2017). Although the corticospinal excitability is modulated by intracortical inhibitory and excitatory circuits in the primary motor cortex (Ni et al., 2011b; Elahi, Gunraj & Chen, 2012), it is not clear which cortical circuits determine the activation difference between ME and MI.

The excitability of various inhibitory and facilitatory intracortical circuits in the human motor cortex can be tested using TMS. Motor evoked potential (MEP) amplitude elicited by a single-pulse TMS is the measure of corticospinal excitability. Short-interval intracortical inhibition (SICI) elicited by a paired-pulse paradigm TMS is the most common and best investigated intracortical inhibitory (Ni et al., 2011b; Dai et al., 2016). It can be elicited when a subthreshold conditioning stimulation suppresses a following suprathreshold test stimulation at interstimulus intervals of 1–5 ms (Ni et al., 2014). Pharmacological studies have suggested that SICI is mediated by type A γ-aminobutyric acid (GABA_A_) receptors (Di Lazzaro et al., 2005). Studies on the human motor cortex using TMS have reported that SICI during MI is significantly reduced, similar to that during ME (Stinear & Byblow, 2004; Leung et al., 2015). These studies raised the possibility that the excitability difference between ME and MI may be related to the activation of the excitatory circuits. To test this possibility, we examined the differences between the aftereffects of ME and MI on corticospinal excitability and SICI. We hypothesized that MI would prompt weaker activation of the excitatory circuit within the primary motor cortex than ME.

## Materials and Methods

### Participants

We recruited 10 healthy young subjects aged 18−28 years (mean age: 22.1 ± 3.14 years; five women and five men) for this study. All subjects were confirmed to be right-handed using the Oldfield Handedness Inventory (Oldfield, 1971). None of the participants had neurological, psychiatric, or other medical disorders, nor did they exhibit any contraindication to TMS. This study was approved by the Human Research Ethics committee of the Shanghai University of Sport (Ethical Application Ref: SUS2014024), and all the subjects provided written informed consent in accordance with the Declaration of Helsinki.

### Motor execution and motor imagery

Subjects were seated in a chair with a fixed headrest. The tasks consisted of either executing or imagining grasp actions of the right hand. For the ME task, participants were required to repeatedly grasp using all the fingers of their right hands. A sensory stimulation cue applied to the right wrist (stimulus intensity of two times the sensory threshold), rhythmically delivered at a frequency of 0.1 Hz using a Digitimer DS7A (Digitimer Ltd, Hertfordshire, UK) constant current stimulator (0.2 ms square-wave pulses) with standard bar electrodes (cathode proximal), was administered to prompt the grasping action. The trial ended when all the fingers were released. The same investigator recorded the duration of ME for all participants by means of a keyboard button. For the MI task, subjects were required to repeatedly imagine, from a first-person perspective, their right hand grasping using all the fingers. The participants were allowed to decide the speed of the imagined action. A sensory stimulation cue (see above) was administered as the prompt. Subjects closed their eyes before beginning the MI task, and opened them at its conclusion. To estimate the duration of the simulated movements, the time during which the participants had their eyes closed was recorded by means of a keyboard button pushed by the same experimenter for all participants. The participants completed one executed movement or one imagined movement every 10 s, for a total of 90 times over the course of 15 min.

To confirm that the participants were able to form mental images with sufficient vividness, they completed the Revised Movement Imagery Questionnaire (MIQ-R) before the intervention (Fourkas et al., 2008). The MIQ-R included four kinesthetic items and four visual items. The difficulty of visualizing a given item was rated using a seven-point scale: a score of 7 indicating the greatest difficulty.

### Experimental design

The first experiment session tested the aftereffects of a period of MI on corticospinal excitability and SICI, while the second assessed the aftereffects of a period of ME on these parameters. Using these two interventional protocols, MI and ME were compared. Each interventional protocol was performed on a separate day, at least two weeks apart, in a random order. Measurements were obtained before each interventional protocol (baseline) as well as 0, 20, and 40 min afterwards (T0, T20, and T40).

### Measurements of corticospinal excitability and SICI

To monitor the changes in corticospinal excitability and SICI after a period of ME or MI, MEPs of the right abductor pollicis brevis (APB) and the first dorsal interosseous (FDI) muscles were recorded after stimulation of their motor cortical representational fields by single-pulse TMS. SICI at interstimulus intervals of 2 ms was tested using a paired-pulse TMS. The test pulse intensity was set at “1 mV,” and the conditioning pulse intensity was set at 70% of the resting motor threshold (Ni et al., 2014).

The MEPs were induced using a Magstim 200 stimulator (Magstim, Dyfed, UK) and a figure-of-eight-shaped coil (the outer diameter of each loop was 9.5 cm) applied to the left primary motor cortex. The coil was held tangentially to the skull, with the handle (approximately perpendicular to the central sulcus) pointing backwards and laterally at an angle of 45° to the sagittal plane. Monophasic pulses, which elicited a posterior-anteriorly directed current in the brain, were used to deliver TMS. For the paired-pulse experiment, two Magstim 200 stimulators were connected to the same coil through a BISTIM module. The APB muscle was selected as the target muscle. The optimal scalp position for inducing MEPs in the right APB muscle was determined by moving the coil in steps of one cm until the largest MEPs were obtained, the location was marked using a pen as the motor hot spot. The MEP amplitude was measured with the intensity of the TMS set to 1 mV: the lowest needed to generate MEPs of >1 mV in at least five out of 10 trials in the target APB muscle when completely relaxed. Resting motor threshold for the APB muscle was defined as the lowest TMS intensity needed to generate MEPs of >50 μV in at least five out of 10 trials when the muscle was completely relaxed.

Surface electromyograms were recorded from the APB and FDI muscles using nine mm diameter Ag–AgCl surface electrodes. The active electrode was placed on the muscle belly, and the reference electrode was placed on the metacarpophalangeal joint of the finger. A ground electrode was placed on the right wrist. The signal was amplified (1,000×), band-pass filtered (20 Hz–2.5 kHz; Matrix 1005; Micromed, Venice, Italy), digitized at a rate of 10 kHz by an analog-to-digital interface (Micro1401; Cambridge Electronics Design, Cambridge, UK), and stored in a computer for offline analysis.

### Data and statistical analysis

Values for kinesthetic and visual items were the total scores of four items in each category. MEP amplitudes were measured peak-to-peak. For SICI measurement, the paired-pulse-induced MEP amplitude was expressed as a percentage of that induced by the test stimulus alone. Values <100% indicate inhibition and values >100% indicate facilitation.

The relationship between ME time and MI time was assessed using Pearson’s correlation test. We performed an independent- sample *t*-test to further compare ME time with MI time. A two-way repeated measures analysis of variance (ANOVA) was used to assess the effects of the interventional protocols and times on the MEP amplitudes and SICI. If the ANOVA showed significant main effects or interactions, post hoc paired *t*-tests with Bonferroni’s correction for multiple comparisons were used to identify the time-points at which the measurements differed among the two interventional protocols. Non-parametric Spearman rank correlation analysis was performed to explore the relationship between the facilitation of corticospinal excitability after MI and the Movement Imagery Questionnaire (MIQ) scores.

The threshold for significance was set at *p* < 0.05. Values are reported as mean ± standard error. SPSS version 17.0 (IBM, Armonk, NY, USA) was used for statistical analysis.

## Results

### Behavioral measures

Table 1 shows the mean values (standard deviation) of the behavioral measures under ME, and MI conditions. The mean scores of the MIQ-R on kinesthetic items (maximum score = 13) and visual items (maximum score = 11) under the MI condition indicated that the participants possessed good MI ability and performed adequately in the MI training.

**Table 1:** Behavioral measures under ME, MI conditions.

	MI	ME
Time course (ms)	1916 ± 152^##^	1354 ± 69
Movement imagery questionnaire		
Kinesthetic items	8.9 ± 2.73	
Visual items	7.7 ± 2.83	

**Notes:**

MI, motor imagery; ME, motor execution.

## *p* < 0.01, comparing ME to MI.

Pearson’s correlation analysis revealed a significant degree of correlation between MI time and ME time (*r* = 0.739, *p* = 0.015), thus confirming that the time course of a mentally simulated movement is positively correlated with its actual execution. Further, a *t*-test was used to assess the difference between the durations of ME and MI. The result revealed that the time of the latter was longer than that of the former (*t*_18_ = −3.36, *p* = 0.003).

### Corticospinal excitability

**Figure 1:**
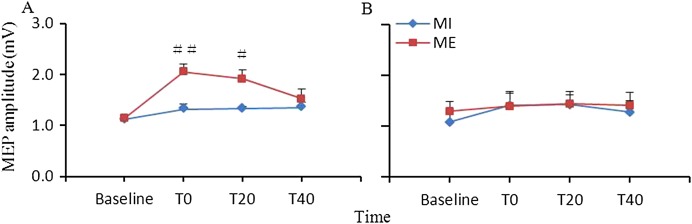
Comparison of the effects of ME and MI on corticospinal excitability. Facilitation of corticospinal excitability after ME was significantly greater at T0 and T20 relative to after MI in the abductor pollicis brevis (A) muscle but not in the first dorsal interosseous (B) muscle (#, *p* < 0.05; ##, *p* < 0.01). Study sites: ME, motor execution; MI, motor imagery.

Figure 1 shows the changes in corticospinal excitability induced by ME or MI. Repeated measures ANOVA revealed significant main effects of interventional protocol (*F*_(1,9)_ = 9.19, *p* = 0.014) and time (*F*_(3,27)_ = 10.99, *p* < 0.001), and a significant interaction between the two main factors (*F*_(3,27)_ = 3.59, *p* = 0.026) in the target APB muscle. Post hoc analysis revealed that the facilitation of corticospinal excitability after ME were greater at T0 and T20 (*p* < 0.01 for T0, and *p* < 0.05 for T20) than those following MI training. For the nontarget FDI muscle, ANOVA revealed that the main effects of interventional protocol (*F*_(1,9)_ = 0.27, *p* = 0.617) and time (*F*_(3,27)_ = 2.17, *p* = 0.115) and the interaction between the time and interventional protocol (*F*_(3,27)_ = 0.33, *p* = 0.803) were not significant.

### Short-interval intracortical inhibition

**Figure 2:**
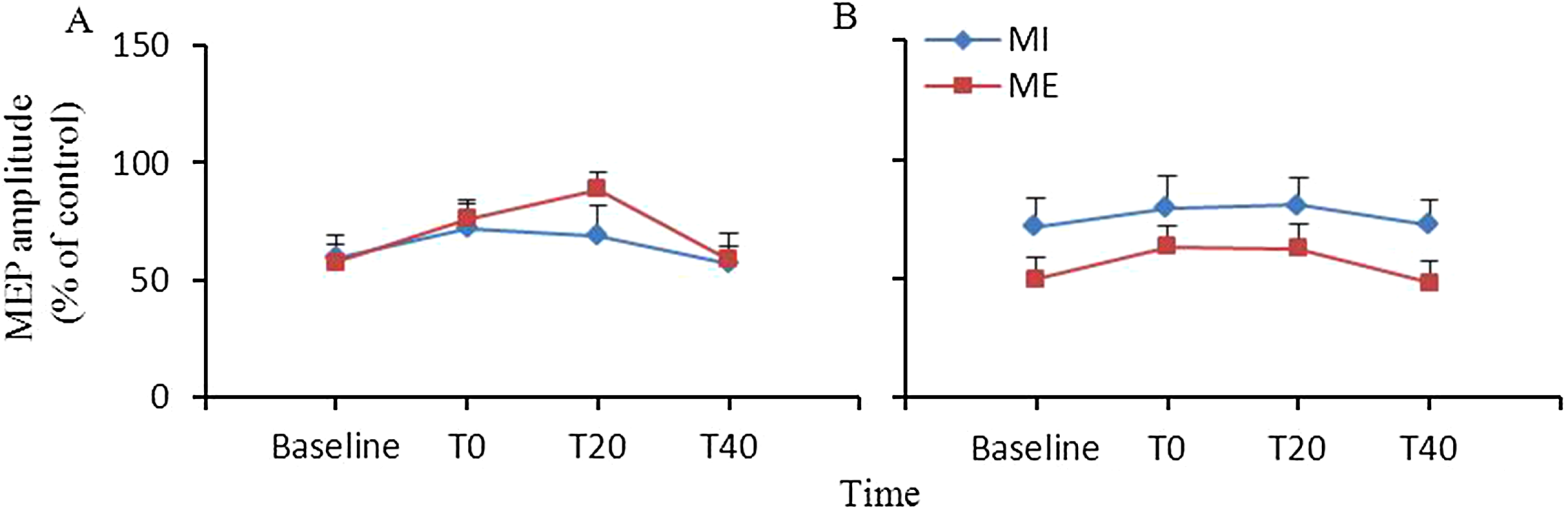
Comparison of the effects of ME and MI on SICI. SICI after ME was not different from that after MI in the abductor pollicis brevis (A) and first dorsal interosseous (B) muscles. Abbreviations: ME, motor execution; MI, motor imagery; SICI, short interval intracortical inhibition.

Figure 2 shows the changes in SICI induced by ME or MI. ANOVA revealed significant main effect of time (*F*_(3,27)_ = 6.86, *p* = 0.001). The main effect of interventional protocol (*F*_(1,9)_ = 1.15, *p* = 0.312) and the interaction between interventional protocol and time (*F*_(3,27)_ = 0.70, *p* = 0.563) were not significant in the target APB muscle. For the nontarget FDI muscle, ANOVA revealed that the main effects of interventional protocol (*F*_(1,9)_ = 2.97, *p* = 0.119) and time (*F*_(3,27)_ = 1.40, *p* = 0.264) and the interaction between the time and interventional protocol (*F*_(3,27)_ = 0.12, *p* = 0.946) were not significant.

### Correlation between behavioral and neurophysiological measures

**Figure 3:**
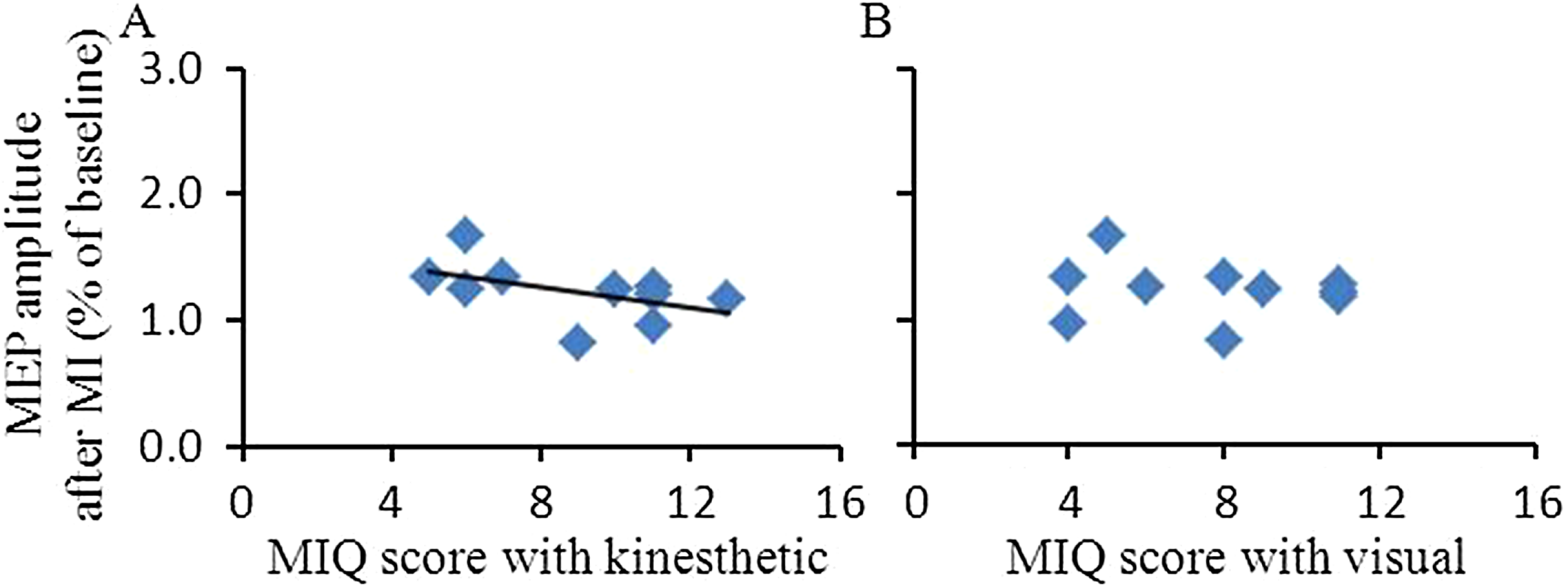
Relationship between MIQ score and MEP amplitude after MI in the abductor pollicis brevis muscle. The abscissas indicate MIQ score with kinesthetic items (A) and with visual items (B). The ordinates indicate the MEP amplitude at T20 after MI. The values were expressed as the percentage of MEP amplitude after the interventional protocol to the MEP amplitude measured before the interventional protocol (baseline). The dark line represents a regression line indicating a positive correlation between MI ability (lower value of MIQ score represents the higher clarity of MI) and intracortical facilitation after MI. Abbreviations: MIQ, movement imagery questionnaire; MI, motor imagery; MEP, motor evoked potential.

We analyzed the correlation between the MIQ scores and the facilitation of corticospinal excitability after MI in the target APB muscle (Fig. 3). Data from all subjects showed that the facilitation of corticospinal excitability at T20 after MI correlated with the MIQ scores for kinesthetic items (*Rho* = −0.646, *p* = 0.044) but did not correlate with MIQ scores for visual items (*Rho* = −0.265, *p* = 0.458).

## Discussion

In this study, we used TMS to examine the differences between the aftereffects of ME and MI on the human motor cortical inhibitory and excitatory circuits. Our novel finding is that the degree of corticospinal excitability (measured with MEP amplitude) after ME was larger than after MI at T0 and T20, but there was no difference in intracortical inhibition (measured with SICI). The results suggest that the excitability difference between ME and MI may be associated with differential activation of the excitatory circuits in the primary motor cortex.

### Parallelism between ME and MI

An internal rehearsal of a motor task without overt physical action, MI plays a critical role in motor skill learning and motor rehabilitation in patients with brain injury (Burianová et al., 2013; Page & Peters, 2014). Prior research has found evidence suggesting parallelism between ME and MI. Several studies involving normal subjects using mental chronometry tasks have shown that the time required to mentally complete a particular movement positively correlates with the time needed to execute the corresponding motor act (Sirigu et al., 1996; Vargas et al., 2004). In agreement with such results, our own findings demonstrated that the time course of a mentally simulated movement positively correlates with its actual execution. Prior studies have provided further support for the parallelism between ME and MI by showing that both processes improved motor performance in athletes and musicians (Holmes & Calmels, 2008; Bassolino et al., 2014). Moreover, many previous neuroimaging studies have demonstrated an overlap of the brain networks activated during the imagination and execution of a motor task, such as the primary motor cortex, pre-supplementary and supplementary motor areas, ventral and dorsal premotor cortices, inferior frontal gyrus, posterior parietal cortex, putamen, ventrolateral thalamus, and cerebellum (Vry et al., 2012; Burianová et al., 2013).

The TMS technique has become a valuable tool for mapping primary motor cortex excitability. Previous studies have used this method to show that the mental simulation of a movement can result in changes in synaptic excitability and plasticity in the primary motor cortex, similar to those induced by actual motor performance, even if the change was greater after ME than after MI. Several studies have shown that MI increases motor cortical excitability (Cengiz & Boran, 2016; Bonassi et al., 2017). Furthermore, motor cortex plasticity can occur during MI, in the absence of actual movement (Pascual-Leone et al., 1995). On the other hand, some studies have demonstrated that decreased intracortical inhibition may contribute to the enhancement of the motor cortex excitability during MI, similar to that during ME (Liang et al., 2011; Leung et al., 2015).

### Difference in intracortical excitation between ME and MI

Although ME and MI share similar components in the motor pathways (Wang et al., 2014), some studies have shown that the time courses and underlying neural elements for MI and ME may not be identical (Burianová et al., 2013; Avanzino et al., 2015). Some neuroimaging studies in healthy subjects have demonstrated that the primary motor cortex and primary somatosensory cortex may show mild activity during kinesthetic-type MI, however, the activity in these areas is typically much greater during ME than during MI (Hanakawa, Dimyan & Hallett, 2008). Other behavioral and neurophysiological studies have shown that MI leads to a lower amount of learning and weaker motor cortical activation than ME (Avanzino et al., 2015; Bonassi et al., 2017). In accordance with these studies, our study also revealed significant differences between ME and MI. In terms of behavior, the time taken to complete the MI tasks was longer than that taken to execute the physical motor tasks. Regarding corticospinal excitability and SICI, the excitability of the primary motor cortex following ME was larger than that following MI intervention in the APB muscle.

One possible interpretation of this difference in the present study is that the subjects did not perform the requested MI task. The following reasons, however, suggest that this explanation should be discarded: (1) the mean scores of MI ability measured under the MI condition indicated that the participants possessed good MI ability, and performed adequately in the MI training; (2) the positive correlation between the MEP amplitudes and MIQ scores for kinesthetic items further confirmed that all subjects used kinesthetic imagery during the MI task; and (3) the direct comparison of pre-intervention with post-intervention effects demonstrated that MI enhanced the excitability of the motor cortex.

It is widely accepted that the balance and interactions between excitatory (e.g., glutamatergic intracortical facilitation) and inhibitory (e.g., GABAergic SICI) circuits determine the final output from the primary motor cortex (Ni et al., 2011a; Dai et al., 2016). Previous studies have shown that both decreased SICI, mediated by GABA_A_ receptor, and increased intracortical facilitation, mediated by *N*-methyl-d-aspartate receptors, may contribute to the enhancement of the motor cortex excitability after ME (Leung et al., 2015; Dai et al., 2016). On the other hand, it has been found that decreased intracortical inhibition during MI is similar to that during ME (Liang et al., 2011). We speculated that the final output differences between ME and MI may be attributed to the differences in the motor cortical excitatory circuit. In support of the above speculation, we observed larger corticospinal excitability after ME than that after MI, but there was no difference in SICI between ME and MI. Therefore, we believe that the final output differences between ME and MI seem not to be rooted in SICI, but rather in MI’s less efficient activation of the excitatory circuits within the primary motor cortex. A possible explanation is the increase of the inhibitory drive over the primary motor cortex originating from many other cortical areas, which prevents ME during mental practice (Guillot et al., 2012). Indeed, a study has demonstrated that the cerebellum has an inhibitory effect on MI (Cengiz & Boran, 2016). Another study has also shown that interactions between supplementary motor area and primary motor cortex are facilitatory during ME and inhibitory during MI (Kasess et al., 2008). Although we considered only grasping movements, we believe that mental practice of the upper limb movements (such as finger, wrist, and arm movements) may share common features that the inhibitory drive over the primary motor cortex originating from many other cortical areas increase, because the voluntary control of movements is often unitary (Brunamonti, Ferraina & Paré, 2012) in all the arm effectors that functionally coordinate (Jeannerod et al., 1995) during reaching and grasping movements.

In our analysis of the FDI muscle, however, the differences between the aftereffects of ME and MI on corticospinal excitability and SICI were non-significant. This may be either because TMS was targeted to the APB muscle or because we recruited an inadequate number of subjects.

A recent study showed that cortical excitability, tested using TMS, increased similarly during voluntary muscular contraction and during MI combined with functional electrical stimulation (Kaneko et al., 2014). This finding highlights that MI combined with non-invasive brain stimulation may be more effective in modulating the increase of intracortical excitatory than MI alone. Therefore, a possible development from our study might be to investigate whether the effect of MI on motor cortex is as effective as that of ME when non-invasive brain stimulation of other cortical areas associated with MI modifies the intracortical excitatory circuit during MI.

## Conclusions

In conclusion, the present study demonstrates that excitability of the primary motor cortex is larger after ME intervention than after MI intervention in the APB muscle. These results suggest that the cortical excitability differences between ME and MI may be related to the activation of the excitatory circuit in the primary motor cortex.

## Supplemental Information

10.7717/peerj.5588/supp-1 Supplemental Information 1 Raw data of intracortical inhibitory and excitatory in the primary motor cortex.

## Additional Information and Declarations

### Data Availability

The following information was supplied regarding data availability: The raw data are provided in the Supplemental File.
